# Obstetric antiphospholipid syndrome: Number 6 - June 2021

**DOI:** 10.1055/s-0041-1732382

**Published:** 2021-07-27

**Authors:** Venina Isabel Poço Viana Leme de Barros, Ana Maria Kondo Igai, André Luiz Malavasi Longo de Oliveira, Marcelo Melzer Teruchkin, Fernanda Andrade Orsi

**Affiliations:** 1 Hospital das Clínicas, Faculdade de Medicina, Universidade de São Paulo, São Paulo, SP, Brazil; 2 Hospital das Clínicas, Faculdade de Medicina, Universidade de São Paulo, São Paulo, SP, Brazil; 3 Hospital Pérola Byington, São Paulo, SP, Brazil; 4 Hospital Moinhos de Vento, Porto Alegre, Rio Grande do Sul, Brazil; 5 Faculdade de Ciências Médicas da Universidade de Campinas, Campinas, SP, Brazil

## Key points

- Antiphospholipid syndrome (APS) is an acquired thrombophilia often associated with adverse obstetric outcomes.
- APS severity depends on the type and complexity of the antibodies. Triple positivity for antiphospholipid antibodies and high antibody titers are commonly associated with a more severe disease.
- The antiphospholipid antibodies described for the diagnosis of the syndrome are: IGG and IGM anticardiolipin, lupus anticoagulant and IGG and IgM antibeta2 GPI.
- The occurrence of venous and/or arterial thrombosis is part of the clinical condition.
- Treatment for APS in pregnancy consists in the use of low molecular weight heparin (LMWH) and low dose aspirin. The dose for anticoagulation depends on the presence or absence of previous thrombosis and the type of obstetric morbidity.
- Patients refractory to anticoagulation treatment may need additional therapies (hydroxychloroquine, prednisone and/or intravenous immunoglobulin).

## Recommendations

- Primary prophylaxis of pregnancy adverse outcomes with low dose aspirin may be considered in asymptomatic antiphospholipid antibody (aPL) carriers who present a high-risk profile.
- Primary prophylaxis of pregnancy adverse outcomes with low dose aspirin and LMWH in prophylactic dose is recommended.
- Patients with previous thrombosis and APS: intermediate or full dose anticoagulation with LMWH and low dose aspirin during pregnancy.
- Patients with serious adverse pregnancy outcomes previously treated with aspirin and LMWH should receive hydroxychloroquine started before pregnancy plus aspirin and LMWH. (evidence level 2C).

## Background

Antiphospholipid syndrome (APS) is a pro-thrombotic and inflammatory condition characterized by thromboembolic events or obstetric complications combined with the presence of at least one antiphospholipid antibody (aPL): lupus anticoagulant (LAC), anticardiolipin (aCL) or anti-β2glycoprotein I (aβ2GP1). 1

## Diagnosis of antiphospholipid syndrome

Antiphospholipid antibody syndrome (APS) is diagnosed when at least one of the following clinical criteria and one of the following laboratory criteria are present: 1

### Clinical Criteria of APS

#### Pregnancy Morbidity

- One or more unexplained death of a morphologically normal fetus >10 weeks of gestation.
- One or more premature delivery of a morphologically normal fetus < 34 weeks of gestation because of severe preeclampsia (PE) or eclampsia (defined according to standard definitions) or recognized features of placental insufficiency.
- Three or more unexplained consecutive miscarriages at <10 weeks of gestation with maternal and paternal factors (such as anatomical, hormonal, or chromosomal abnormalities) excluded.

#### Vascular thrombosis

One or more clinical episode of arterial, venous, or small-vessel thrombosis.

- Thrombosis must be objectively confirmed.
- If histopathological confirmation is used, thrombosis must be present without inflammation of the vessel wall.

### Laboratory criteria

1. Positive lupus anticoagulant (LA) in plasma on two or more occasions at least 12 weeks apart.
2. Positive anticardiolipin (aCL) antibody of IgG and/or IgM isotype in serum or plasma at medium or high titer (i.e. >40 GPL or MPL, or >the 99th percentile) on two or more occasions, at least 12 weeks apart.
3. Positive anti-b2 glycoprotein-I antibody of IgG and/or IgM isotype in serum or plasma (titers above the 99th percentile) on two or more occasions at least 12 weeks apart.

**Figure 1.**
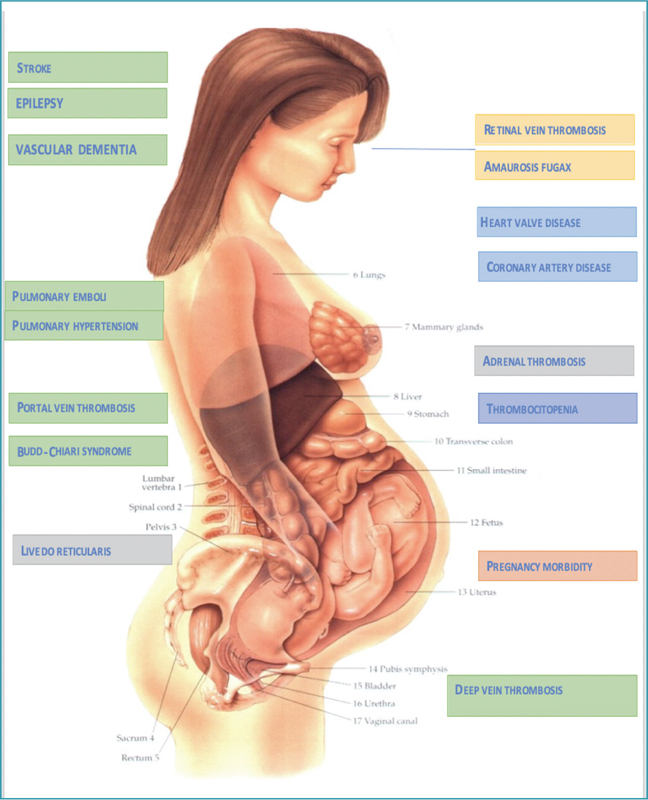
Clinical manifestations of antiphospholipid syndrome in women

Antiphospholipid syndrome can lead to a wide spectrum of thrombotic complications, such as venous thromboembolism (VTE), venous thrombosis in unusual sites, and arterial and capillary thrombosis, which are highly susceptible to recurrence. 2 There are several more symptoms and other organs can be involved, partly noncriteria APS manifestations ( Figure 1 ).

Obstetric complications are unexplained recurrent miscarriages (gestational age [GA] < 10 weeks), death of a morphologically normal fetus, premature birth (GA < 34 weeks) due to preeclampsia, eclampsia, HELLP syndrome or intrauterine growth restriction (IUGR). 1 3

Although antiphospholipid antibodies are at the basis of both thrombotic and obstetric manifestations of APS, different mechanisms are associated with each APS variant. 4 While a pro-thrombotic state is the hallmark of thrombosis in APS, obstetric APS is characterized by defective placentation due to inflammation, activation of the complement system, hypercoagulability and abnormalities in vascular remodeling of the uterine vessels, which may not be related to thrombotic lesions. 4 Although thrombosis and obstetric manifestations are distinct variants of APS, most patients present with both APS complications. 5

Antiphospholipid syndrome, either primary or associated with systemic lupus erythematosus (SLE), can pose several problems to women's health in terms of contraception, reproduction, and menopause treatment. Patient care in daily medical practice can be compromised by heterogeneous clinical presentation and available therapy approaches. The aim of this review and position paper is to provide useful recommendations on the management of women with APS for the medical community.

## Laboratory diagnosis of APS

Antiphospholipid antibodies are widely used as diagnostic markers of antiphospholipid syndrome (APS). The following antibodies and titers are considered laboratory criteria for APS diagnosis: i) positive LAC; ii) IgG or IgM aCL at medium or high titer (>40 GPL/MPL or >99th percentile); iii) IgG or IgM aβ2GP1 at medium or high titer (>99th percentile). At least one of laboratory criteria most be present on two or more occasions, at least 12 weeks apart. 1

Besides being used for diagnosis, the profile of these antibodies may also play a role in the clinical presentation of the disease. Miyakis et al. 1 suggested that APS should be categorized according to aPL positivity, as type I (one positive aPL), type IIa (LAC present alone), type IIb (aCL present alone) and type IIc (aβ2GP1 present alone). Recently, Pengo et al. 6 suggested that the positivity for the three aPL antibodies, known as triple positivity, was an independent risk factor for thrombosis in aPL asymptomatic carriers. Clinical studies suggest that LAC positivity alone, double, and triple positivity are associated with high risk of APS complications ( Table 1 ).

**Table 1.** Definitions of high-risk and low-risk aPL profile

High-risk aPL profile	Low-risk aPL profile
Persistently positive lupus anticoagulant (measured according to ISTH guidelines), or	Isolated aCL or antibeta2 glycoprotein I antibodies at low/medium titers, particularly if transiently positive
Double aPL positivity (any combination of lupus anticoagulant, aCL antibodies or antibeta2 glycoprotein I antibodies), or	
Triple aPL positivity, or	
Persistently high aPL titers	

ISTH International Society of thrombosis and Hemostasis; aCL anticardiolipin; aPL antiphospholipids

Source: Modified from: Tektonidou et al. (2019). 7

## Prevention and treatment of obstetric complications: how to manage these patients?

### Obstetric complications in asymptomatic aPL carriers

The first evidence linking aPL positivity with adverse pregnancy outcomes emerged in the early 90's. In a prospective observational study, Lynch et al. 8 evaluated 389 first time pregnant women, of whom 95 (24%) presented with positive aPL. During follow-up, fetal loss was observed in about 16% of aPL carriers and 6.5% of aPL negative patients, which yielded a 2.5 times higher risk for fetal loss in aPL carrier women than in non-aPL. 8 These results were confirmed by cohort and case-controls studies later evaluated in a meta-analysis that revealed LA carriers were 2 to 4 times more likely to develop late placenta-mediated adverse pregnancy outcomes, such as preeclampsia, IUGR and late fetal loss. 9

The most beneficial approach to prevent obstetric complications in aPL carrier women has not yet been defined, as evaluation of clinical data on primary prophylaxis of obstetric complications are scarce and based on observational data or low numbers of cases, which renders low-quality evidence. A randomized study that included 19 asymptomatic patients with positive aPL showed no benefits in using low dose aspirin (85 mg OD) compared to usual care, even though the number of adverse events such as fetal loss and IUGR were low and the study was underpowered to show actual differences between treatments. 10 More recently, in a retrospective study, Del Ross et al. 11 described the effect of low dose aspirin (100mg OD) on the outcomes of 139 pregnancies in aPL positive women not fulfilling criteria for APS. The risk of miscarriage, prematurity and IURG was similar between women who used or not low dose aspirin, and the frequency of live birth was high (above 92%) regardless of the treatment. 11

Although no clinical evidence supports primary prophylaxis of obstetric complications among aPL carriers, available studies have not evaluated prophylaxis in patients with high-risk profile (LAC positivity alone, double, and triple positivity) in which the risk of adverse outcomes may justify early medical intervention. For that, experts on APS from the European League Against Rheumatism (EULAR) recently agreed that it is reasonable to consider using low dose aspirin (75 – 100mg OD) in asymptomatic pregnant women with high-risk aPL profile ( Table 1 ) and no previous history of thrombosis or obstetric complications. 7 A summary of treatments according to the clinical profile of patients can be seen in table 2 .

**Table 2.** Management of pregnant women with antiphospholipid antibodies or APS

Clinical manifestations	Treatment	Evidence
Persistent presence of aPL without adverse pregnancy outcomes or thrombosis	Close monitoring of fetus and mother during pregnancy with or without LDA treatment.	No studies performed on APS. Risk factors to be considered: age >35 y, presence of autoimmune diseases, chronic hypertension.
Persistent positivity for antiphospholipid antibodies and history of recurrent first trimester pregnancy loss	LDA with prophylactic LMWH	Low-quality randomized controlled trials
Previous history of placenta-mediated complications	LDA with prophylactic LMWH	Low-quality randomized controlled trials
Patients with thrombotic APS (venous or arterial)	LDA and intermediate-dose or full-dose LMWH	Based on one prospective observational study
Anticoagulation in postpartum period and APS	LMWH thromboprophylaxis for six weeks postpartum if previous thrombosis; two weeks postpartum if no previous thrombosis or additional risk factors.	Based on case-control studies and cohort studies

APS – antiphospholipid syndrome, aPL- antiphospholipid antibodies; LDA – low dose aspirin; LMWH – low molecular weight heparin. Low dose aspirin -80-150mg/day

Source: Adapted from: Czwalinna and Bergmann (2020). 12

### Obstetric complications in APS patients: is everything solved?

In a large cohort of 1000 patients with APS (many of whom had SLE), which evaluated 188 pregnancies over ten years, the absolute risks for fetal loss, IUGR and prematurity were high, 16%, 26% and 48%, respectively. 5 Interestingly, although the proportion of early pregnancy loss has decreased (from 35% to 16%) combined with higher chances of live birth (from 47% to 73%) over the ten-year period of follow-up, the risk of a live birth with prematurity or IUGR remained extremely high (above 30%). 5 This observation highlights that while the current strategies seem efficient to prevent miscarriages in APS women, there are unmeet clinical needs in the treatment of APS-related late pregnancy complications.

The first randomized studies that evaluated treatment approaches towards the prevention of recurrent miscarriages were performed in the late 90's and early 2000's. In a randomized clinical trial, Rai et al. 13 demonstrated that the combination of low dose aspirin and low molecular weight heparin was superior to low dose aspirin alone in preventing miscarriages among APS patients. The proportion of live births was roughly two times higher in the group receiving low dose aspirin plus low molecular weight heparin (71%) when compared to the group receiving low dose aspirin alone (42%). 13 Although a subsequent randomized trial failed to demonstrate differences between low dose aspirin alone and low dose aspirin plus low molecular weight heparin therapies in pregnant women with APS and recurrent miscarriages, 14 the study had several methodological issues as the inclusion of pregnant women after the 12th week of gestation and high proportion of protocol violations (25% of women switched study arms). Further observational studies and meta-analysis confirmed the superiority of low dose aspirin and low molecular weight heparin for the prevention of early pregnancy losses. An observational study evaluating 176 aPL/APS women with recurrent miscarriages and 517 women with unexplained miscarriages demonstrated that the chance of a live birth was increased by more than twofold in aPL/APS women using low dose aspirin and low molecular weight heparin as compared to those using low dose aspirin alone. 15 Any treatment effect of low dose aspirin or low dose aspirin plus low molecular weight heparin was observed in pregnant women with previous unexplained miscarriages. 15 In a meta-analysis of five randomized controlled trials, the use of low dose aspirin plus low molecular weight heparin was overall associated with higher rates of live birth than low dose aspirin alone, although there was no difference between the two treatment strategies with regard to the rate of premature labor and preeclampsia. 16

Treatment strategies to prevent late pregnancy complications were recently evaluated by the FRUIT trial. In this study, 32 APS pregnant women with a history of preeclampsia, eclampsia or HELLP syndrome in previous pregnancies were randomized to receive low dose aspirin plus low molecular weight heparin or low dose aspirin alone during the current pregnancy. The study demonstrated that the absolute risk of these hypertensive disorders of pregnancy was not reduced by the use of low dose aspirin plus low molecular weight heparin, as compared to low dose aspirin alone, suggesting that low dose aspirin plus heparin does not add treatment benefits to standard low dose aspirin alone in terms of preventing late pregnancy complications. 17

Taking into account the evidence that the use of low dose aspirin plus low molecular weight heparin is superior to low dose aspirin alone in reducing miscarriages but not late pregnancy adverse outcomes in APS, the use of low dose aspirin plus low molecular weight heparin during the entire pregnancy is suggested to prevent recurrent miscarriage and fetal loss among APS women. In APS women with previous premature birth due to preeclampsia, eclampsia, HELLP syndrome or placental insufficiency, either low dose aspirin plus low molecular weight heparin or low dose aspirin alone could be used to prevent the recurrence of these late pregnancy complications. 7

## Management of refractory obstetric APS

A recently published European survey of 1000 consecutive cases of obstetric APS revealed that to date the proportion of fetal loss is still very high (at 27%) among APS women 18 and recurrent miscarriage is the most frequent poor outcome, even though therapy strategies to treat these patients have been improved in recent years.

Indeed, the risk of treatment failure is an important issue that may affect more than 20% of patients with obstetric APS and some risk factors associated with treatment failure have been identified. In a large case-control multicenter study, treatment failure was more likely to occur among women with SLE or other autoimmune diseases, history of both thrombosis and pregnancy complications and triple positivity for aPL. 19 A recent multicenter cohort study confirmed that the presence of autoimmune disease, complement consumption and previous thrombosis were risk factors for the occurrence of adverse pregnancy outcomes in APS, regardless of the treatment approach used to prevent these outcomes. 20

Various clinical treatments for the treatment of refractory obstetric APS have been described, such as hydroxychloroquine, glucocorticoids, immunoglobulin and plasmaphereses. 21 22 23 24 However, these treatments were described mainly in case-series studies and there is no robust clinical evidence to support the use of these therapies. The best available evidence in this regard come from two recent cohort studies. Two multicenter retrospective studies have demonstrated the benefits of adding hydroxychloroquine to conventional treatment in order to increase live birth rates in refractory obstetric APS cases. 25 26 A very recent study, published in 2020, showed that combinations of low dose aspirin with low molecular weight heparin at therapeutic dose could improve pregnancy outcomes in patients with severe pregnancy complications. 26 However, these studies suffer from confounding by indication bias, because the treatment strategy was not randomly assigned but chosen based on the clinical features of patients. Moreover, alternative treatments were mostly compared with historical data, which can result in numerous information biases. Taking together, the available data point towards a possible effect of hydroxychloroquine on improving pregnancy outcomes in APS. To confirm this suspicions, two randomized clinical trials are being performed to evaluate the impact of hydroxychloroquine in addition to standard therapy in the improvement of pregnancy outcomes in women with obstetric APS, 18 27 but the results of these studies are not yet available.

To date, current suggestions for the treatment of obstetric APS refractory to low dose aspirin plus prophylactic low molecular weight heparin are based on experts' opinion only. Possible therapeutic approaches include: low dose aspirin plus therapeutic dose of low molecular weight heparin, hydroxychloroquine, low dose of glucocorticoids during the first trimester of pregnancy and immunoglobulin. 7

## Patients with APS and pregnancy morbidity are at greater risk for thrombosis?

The following risk factors were associated with a greater risk for having a first thrombosis after a pregnancy morbidity: 28

- younger age at diagnosis of Ob-APS
- additional cardiovascular risk factors
- superficial vein thrombosis
- heart valve disease
- multiple aPL positivity

## Catastrophic antiphospholipid syndrome and pregnancy: a diagnosis that should not be missed

Catastrophic antiphospholipid syndrome (CAPS) is a rare but life-threatening condition that may be precipitated by pregnancy. The condition can be hard to diagnose since it mimics other pregnancy-associated thrombotic microangiopathies. Accurate and timely diagnosis is critical for effective treatment. 29 Criteria for CAPS include multi-organ thrombosis over a one-week period of time that affects at least three organs or tissues. However, these are meant to be guidelines used for classification purposes rather than definitive clinical care. The condition is rare, accounting for less than 1% of APS cases, but can be life threatening and pregnancy may be a trigger. Besides pregnancy, precipitating factors are present in most cases and include infections, surgery, malignancy, contraceptives and drugs. Pregnancy is the precipitating factor in about 8% of cases. 30

Pregnancy-related CAPS occurs in younger individuals than those who are not pregnant. In addition, CAPS is more likely to present de novo in pregnancy (48.2%) compared to non-pregnancy (26.3%). Pregnancy-related CAPS also is relatively more likely to be associated with liver involvement and previous pregnancy loss. The differential diagnosis includes other thrombotic microangiopathies, many of which are associated with (or specific to) pregnancy. Conditions sharing many features with CAPS include preeclampsia, HELLP syndrome, hemolytic-uremic syndrome (HUS), thrombotic thrombocytopenic purpura (TTP), lupus flare, disseminated intravascular coagulation, and acute fatty liver of pregnancy (AFLP). All these conditions are characterized by microangiopathic hemolytic anemia, thrombocytopenia and potential malignancy.

The treatment basis is therapeutic anticoagulation. Immunosuppression, supportive treatment and removal or treatment of any precipitating factors are recommended. Other treatments focus on immunosuppression. First-line treatment usually include corticosteroids, although efficacy is uncertain. In addition to corticosteroids, intravenous immunoglobulin (IVIG) or plasma exchange is used for additional immunosuppression and treatment. Optimal dosing is uncertain but typically 0.4 g/kg per day for three–five days. In some centers, they proceed with immediate delivery if gestational age is ≥34 weeks' gestation. At earlier gestational ages, proceed to delivery if the patient does not respond to treatment after a reasonable time interval (e.g. 24–48 hours) or if fetal status is compromised. 29 Rituximab is a chimeric monoclonal antibody against CD20 positive B cells. Rituximab has been reported to be useful in improving APS (in patients without CAPS) in uncontrolled cases series. Outcomes were good (75%) in 20 patients with CAPS treated with rituximab. 31 Another monoclonal antibody, eculizumab, is specific for complement protein C5. There are several ongoing studies evaluating the use of eculizumab in APS and CAPS. Meanwhile, the medication should be reserved for refractory cases due to high cost. 32

## Final considerations

- Thrombotic and obstetric APS are two different variants of the same syndrome.
- In pregnant women with APS, the proportion of fetal loss and late obstetric complications are about 15-35% and 5%, respectively.
- Primary prophylaxis of pregnancy adverse outcomes with low dose aspirin may be considered in asymptomatic aPL carriers who present with a high-risk profile.
- Conventional treatment for preventing obstetric complications consists of the association of low dose aspirin and low molecular weight heparin at prophylactic doses.
- Adequate treatment options for refractory cases are not established, although treatment strategies using hydroxychloroquine, prednisone, immunoglobulin and plasmaphereses have been described in case-series studies and the use of hydroxychloroquine seems to be the most promising therapy for refractory obstetric APS.

National Specialty Commission for Venous Thromboembolisms of the Brazilian Federation of Gynecology and Obstetrics Associations (FEBRASGO)

President:

Venina Isabel Poço Viana Leme de Barros

Vice-President:

André Luiz Malavasi Longo de Oliveira

Secretary:

Paulo Francisco Ramos Margarido

Members:

Ana Maria Kondo Igai

Cristiano Caetano Salazar

Denis Jose Nascimento

Eduardo Zlotnik

Egle Cristina Couto

Eliane Azeka

Fernanda Andrade Orsi

Joaquim Luiz de Castro Moreira

Marcelo Melzer Teruchkin

Marcos Arêas Marques

Mônica Cristina da Costa Drago Souza

Valeria Doria
